# Mitral Valve-In-Valve: Defining the Indication Limits by *in vitro* Hydrodynamic Tests in a Brazilian Transcatheter Prosthesis

**DOI:** 10.21470/1678-9741-2020-0477

**Published:** 2021

**Authors:** Thiago Vila Nova, Caio Cardoso, Ademir Braz, Honório Palma, Diego Gaia

**Affiliations:** 1 Department of Cardiovascular Surgery, Federal University of São Paulo, São Paulo, São Paulo, Brazil.

**Keywords:** Heart Valve Prosthesis Implantation, Hydrodynamic, Mitral Valve, Reoperation, Hydrodynamics

## Abstract

**Introduction:**

Reoperations in cardiac surgery represent a clinical challenge, particularly because of the higher rate of perioperative morbidity and mortality. Mitral valve reoperation owing to bioprosthesis dysfunction, transcatheter treatment with a prosthesis implantation over the prosthesis has emerged as an alternative, especially for patients with a previous approach. In this study, we analyzed the hydrodynamic behavior of transcatheter prosthesis implantation in conventional mitral bioprostheses through hydrodynamic tests and produced a recommendation for the size of transcatheter valve most adequate for valve-in-valve procedure.

**Methods:**

Mitral bioprostheses were attached to a flow duplicator and different combinations of transcatheter prostheses were implanted inside. The equipment simulates the hydrodynamic behavior of the valves submitted *in vitro* and determines transvalvular pressures and flow parameters.

**Results:**

All tests could be performed. Better hydrodynamic performance occurred for transcatheter prostheses 1 mm smaller than bioprostheses, except for the 27-mm bioprostheses. Effective valve areas (cm²) and transvalvular gradients (mmHg) were, respectively: Bioprosthesis × Inovare: 27 × 28 mm: 1.65 and 5.95/29 × 28 mm and 31 × 30 mm: 2.15 and 3.6.

**Conclusion:**

The mitral valve-in-valve implantation proved to be feasible *in vitro*. The use of 27-mm bioprostheses should be judicious, with preference for a 26-mm transcatheter valve. In the 29 and 31-mm bioprostheses, the implantation was very satisfactory, with good effective valve areas and transvalvular gradients, with preference for smaller transcatheter valves.

**Table:** 

Abbreviations, acronyms & symbols	
ΔP	= Mean transvalvular gradient
EOA	= Effective orifice area
ID	= Internal diameter
SD	= Standard deviation

## INTRODUCTION

Surgical treatment of the mitral valve is the only alternative in conditions of symptomatic severe mitral insufficiency or stenosis. Replacement of the diseased valve with a prosthesis may be necessary, especially when repair is not feasible. Two types of substitutes can be used, namely bioprostheses and mechanical prostheses ^[1]^. The operation is a standardized treatment, with good results over the past decades, allowing reverse remodeling of the cardiac cavities, recovery of ventricular function and remission of symptoms. Biological prostheses are becoming increasingly preferred over mechanical valves because of the lower rate of thrombotic complications ^[2]^. Unfortunately, these biological leaflet valves (porcine or bovine) have limited durability and an estimated failure within 10 to 20 years, resulting in a population of high-risk patients requiring a new valve prosthesis owing to structural deterioration of the previous bioprosthesis ^[3]^. Degenerative processes of the biological tissue and calcification are the most common complications that determine its limited durability. It occurs due to repetitive stress on heterologous pericardial cusps that causes damage to type I collagen and loss of extracellular matrix glycosaminoglycans, resulting in less elastic force, favoring the retraction of the leaflets, the appearance of shear stress on the surface of the leaflets and facilitating calcification ^[4]^.

Many patients with mitral prosthetic structural deterioration have multiple other comorbidities and risk factors, such as advanced age, New York Heart Association (NYHA) functional classification, number and type of previous operations, cardiac rhythm and character of the operation, may also increase the risk of the procedure. As a consequence, the combination of these factors may lead to contraindication of the procedure, leaving the individual with significant symptoms and risk of death ^[5]^. Recently, several authors have reported the use of transcatheter technology with the objective of reducing the morbidity and mortality associated with conventional reintervention in high-risk patients ^[6]^. The procedure of releasing the transcatheter prosthesis into a bioprosthesis with dysfunction is known as "valve-in-valve" ^[6]^. Advances in the technique have provided safer implants with progressively better results ^[7]^. However, multiple limitations have been reported and information regarding their efficacy, safety, and limitations needs to be expanded ^[8]^.

One of the main limitations of the valve-in-valve implant is the need to determine the adequate valve size to be implanted within the bioprosthesis with dysfunction to balance the risk of migration of the prosthesis, as it is fixed by radial force ^[9]^. In addition, inadequate opening of the transcatheter prosthesis may determine the inadequate excursion of the valve leaflets and, consequently, change the stress on the leaflets, with a reduction in their durability and a reduction in the effective orifice area ^[9]^. Thus, determining the best combination between Brazilian conventional biological mitral prostheses and Brazilian transcatheter prosthesis is essential to improve the indication of the procedure in our country.

In the present study, we aimed to analyze the hydrodynamic behavior of Braile Inovare (Braile Biomédica, São José do Rio Preto, Brazil) transcatheter implant in conventional biological mitral prostheses using hydrodynamic tests. We also aimed to produce a recommendation guide of the most suitable transcatheter valve size for each mitral bioprosthesis.

## METHODS

### General Specifications

This study was conducted after approval by the Research Ethics Committee of the Federal University of São Paulo (No. 4097111215) for studies that do not involve humans or vertebrate animals.

The Pulse Duplicator from ViVitro Labs Inc. (Victoria, British Columbia, Canada) was used at the Braile Biomédica Research Laboratory, São José do Rio Preto, São Paulo, Brazil, for the performance test of the heart valves. It consists of a 2-chamber acrylic system (simulating the atrium and ventricle), pressure transducers, electromagnetic flow meters, a heat exchanger, a centrifugal pump, a data acquisition system, and software for interpretation and presentation of results (LabVIEW), which allows testing of prosthetic devices through the generation of pulse waves and flows.

The tests followed the specifications stipulated in the duplicator’s manual. In addition, cardiac output (L/min) and mean arterial pressure (mmHg) followed the standards established for cardiac prosthetic valve testing by the Food and Drug Administration and International Organization for Standardization (resolution number 5840), representing 5 L/min and 100 mmHg, respectively. Measurements were performed with heart rate simulation of 70, 80, 90, 100, 110, and 120 beats per minute, according to the standardization quoted above.

The apparatus aims to simulate the *in vivo* hydrodynamic behavior to which the valves are subjected and, in this way, to determine the transvalvular pressure and flow parameters through the sensors. The complexes formed by the valves in the equipment were immersed in a 0.9% sodium chloride solution and 1% benzyl alcohol, looking for viscosity close to the blood (between 4 and 5 millipascal per second).

The pressure and flow data through the valve were detected and based on these data, the transvalvular pressure gradient and the effective orifice area were calculated. For each proposed test, 10 bioprosthesis-transcatheter valve sets were repeated. Each set was tested 3 successive times. Thus, considering each simulated heart rate, 180 values were obtained for both the transvalvular gradient and the effective orifice area.

### Mitral bioprostheses

**Fig. 1:**
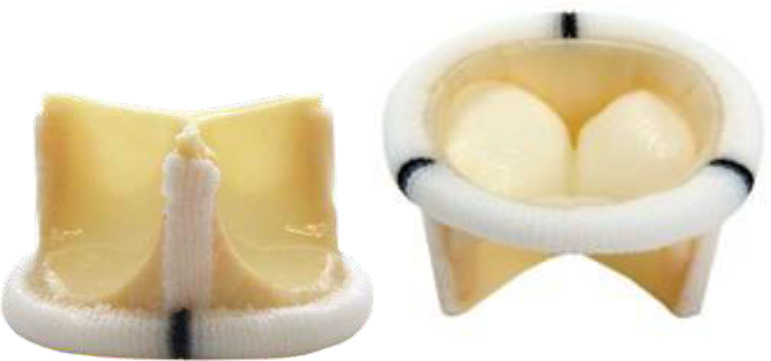
Conventional Braile mitral bioprosthesis.

Braile Biomedical bovine pericardial mitral bioprostheses were used in the study. The prosthetic device is made on a polyacetal support coated with bovine pericardium, with which the 3 cusps are mounted. The base of the bioprosthesis ring is reinforced with stainless steel wire, which allows fluoroscopic identification. The pericardium of the bioprostheses is treated with glutaraldehyde and preserved with 4% formaldehyde. The nominal sizes available for mitral bioprostheses in this study were 27, 29 and 31 mm, which correspond with the sizes most commonly used in mitral valve replacements (Figure 1).

Significant data for subsequent analyses are the diameters of bioprostheses (Table 1). The nominal size corresponds to the internal diameter of the bioprosthesis, which is important for the conventional surgical implantation. The external diameter corresponds to the total diameter of the bioprosthesis, including the cuff, where the fixation of the points of the mitral annulus to the valve prosthesis occurs. Finally, the true internal diameter (true ID), which represents the internal disc diameter of the space occupied by the pericardial leaflets, deserves attention. Such data directly interfere in the effective orifice area and possible transvalvular gradient, which is relevant mainly in the valve-in-valve concept.

**Table 1:** Technical data of the Braile Biomedical bovine pericardial mitral bioprostheses, standing out the true ID, compared with the internal diameter, nominal size and external diameter.

Mitral bioprosthesis, mm	True ID, mm	Internal diameter, mm	External diameter, mm
27	22	27	31
29	24	29	33
31	26	31	35

ID=internal diameter; mm=millimeters

### Transcatheter valves

**Fig. 2:**
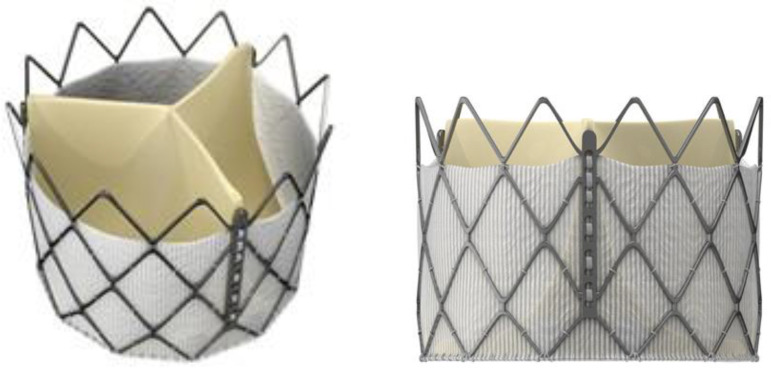
Braile Inovare prosthesis.

To prepare the valve-in-valve, the Braile Inovare prosthetic device was used. Inovare is a valve for transcatheter application, which is made from a single bovine pericardium leaflet (also treated with conventional bioprostheses), with the construction of 3 concentric cusps and fixed in a chromium-cobalt stent structure, internally coated with polyester. Such features allow a wide effective orifice area and effective radial force, which allows the fixation of the valve to the structure of the bioprosthesis with dysfunction. Inovare is available in nominal sizes of 20, 22, 24, 26, 28, and 30 mm. In this study, the 26, 28, and 30 mm sizes were used (Figure 2).

As shown in the bioprosthesis, the external, internal and true ID diameters are fundamental data, especially for the use of transcatheter valves in the valve-in-valve. Due to its preparation and fixation of the cusps in the chromium-cobalt stent structure, Inovare presents a wide true ID proportionally to the conventional bioprosthesis, as shown in Table 2. The nominal size of the transcatheter valve prosthesis corresponds to the external diameter.

**Table 2:** Technical data of the Inovare Braile Biomédica transcatheter valves, standing out the real internal diameter (true ID) compared with the internal diameter, nominal size and external diameter.

Inovare, mm	True ID, mm	Internal diameter, mm	External diameter, mm
26	23	24	26
28	25	26	28
30	27	28	30

ID=internal diameter; mm=millimeters

### Valve-in-valve tests

Biological mitral prostheses of bovine pericardium with a diameter of 27, 29, and 31 mm (Braile Biomédica) were fixed in a stand of the Pulse Duplicator and ViVitro equipment. Within the bioprosthesis, prostheses for transcatheter application with diameters of 26, 28, and 30 mm were implanted, as described below.

**Fig. 3:**
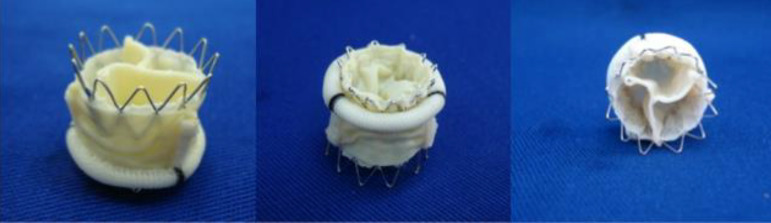
Image showing the structure composed by the overlapping of a conventional prosthesis (27 mm) and a transcatheter prosthesis (26 mm).

The allocation of the transcatheter valve was performed as usual in the transapical approach *in vivo*, with specific device crimping in a valvoplasty balloon of adequate size for each prosthesis and then expanded inside the bioprosthesis, as shown in Figure 3.

To perform the tests with each bioprosthesis diameter, a transcatheter valve with a nominal diameter 1 mm larger and 1 mm smaller than the diameter of the biological valve was implanted (e.g., in a 27-mm mitral bioprosthesis, a 26-mm and a 28-mm transcatheter valve were used). Each test was performed individually. An exception was made for the 31-mm bioprosthesis, with the transcatheter prosthesis tested with the 30-mm, but not the 32-mm transcatheter prosthesis.

The choice of nominal sizes 1 mm above and below the nominal size of the bioprosthesis was the result of considerations between the internal diameter of the bioprosthesis and the external diameter of the transcatheter valve, which, because it is an expandable balloon, requires oversizing. Analyzing these diameters, it was observed that, requiring a radial force for anchoring the transcatheter valve, an oversize of 10% to 20% of the transcatheter prostheses was chosen in relation to the bioprosthesis (Table 3). Thus, the sets of tests were as mentioned in the previous paragraph.

**Table 3:** Oversizing between the true ID of the bioprosthesis and the external diameter of the transcatheter valve.

Bioprosthesis, 27 mm	Oversizing
Inovare, 26 mm	18%
Inovare, 28 mm	27%
Bioprosthesis, 29 mm	Oversizing
Inovare, 28 mm	16%
Inovare, 30 mm	25%
Bioprosthesis, 31 mm	Oversizing
Inovare, 30 mm	15%

mm=millimeters

The statistical method used in the study was Student's *t*-test for continuous variables, to evaluate the most suitable transcatheter valve option for the bioprosthesis test, specifically for the effective orifice area and the transvalvular gradient.

## Results

It was possible to perform the tests in all groups, and there was no migration of the prosthesis in any of the assemblies. In all tests, the estimated cardiac output was adequate (between 4.90 and 5.11 L/min), in simulated heart rates between 70 and 120 beats per minute. With these tests, data obtained included an effective orifice area (EOA, cm²) and mean transvalvular gradient (ΔP, mmHg).

### Valve-in-valve

The results of EOA and ΔP are shown below for each bioprosthesis tested, considering the various transcatheter valve sizes proposed.

### Valve-in-valve in 27-mm bioprosthesis

Regarding the 27-mm mitral bioprosthesis, when comparing the 26-mm transcatheter valve implant with the 28-mm implant, we observed an EOA of 1.63 cm² (SD±0.05) and 1.1 cm² (SD±0.00), and a mean ΔP of 5.95 mmHg (SD±0.10) and 11.75 mmHg (SD±0.28), respectively.

Student's *t*-test showed a statistically significant difference (*P*<0.05) between the EOA (*P*=0.001) and ΔP (*P*=0.001) values, comparing the 2 transcatheter valves when used in 27-mm bioprosthesis (Table 4).

**Table 4:** Values found with the 27-mm bioprosthesis and the 26-mm and 28-mm transcatheter valves.

Hydrodynamic tests: Bioprothesis 27 mm × Inovare 26 mm and Inovare 28 mm				
	**EOA (cm^2^)**		**ΔP (mmHg)**	
**Frequency**	**Inovare 26 mm**	**Inovare 28 mm**	**Inovare 26 mm**	**Inovare 28 mm**
70	1.6	1.1	6.1	12.2
80	1.6	1.1	6.0	11.9
90	1.7	1.1	5.9	11.8
100	1.7	1.1	5.8	11.6
110	1.6	1.1	6.0	11.6
120	1.6	1.1	5.9	11.4
Standard deviation	0.05	0.00	0.10	0.28
Mean	1.63	1.1	5.95	11.75
*P*-value	0.001		0.001	

ΔP=mean transvalvular gradient; cm^2^=square centimeters; EAO=effective orifice area; mm=millimeters; mmHg=millimeters of mercury

### Valve-in-valve in 29-mm bioprosthesis

In the 29-mm mitral bioprosthesis tests, the 28-mm and 30-mm transcatheter valves were used, with a mean EOA of 2.18 cm² (SD±0.04) and 1.73 cm² (SD±0.05), and a mean ΔP of 3.61 mmHg (SD±0.13) and 6.35 mmHg (SD±0.05), respectively. When comparing such values using the Student's *t*-test, there was a statistically significant difference for both EOA (*P*=0.001) and ΔP (*P*=0.001), as shown in Table 5.

**Table 5:** Values found with the 29-mm bioprosthesis and the 28-mm and 30-mm transcatheter valves.

Hydrodynamic tests: Bioprosthesis 29 mm × Inovare 28 mm and Inovare 30 mm				
	**EOA (cm^2^)**		**ΔP (mmHg)**	
**Frequency**	**Inovare 28 mm**	**Inovare 30 mm**	**Inovare 28 mm**	**Inovare 30 mm**
70	2.2	1.8	3.6	6.4
80	2.2	1.7	3.6	6.3
90	2.2	1.8	3.6	6.3
100	2.2	1.7	3.8	6.3
110	2.2	1.7	3.4	6.4
120	2.1	1.7	3.7	6.4
Standard deviation	0.04	0.05	0.13	0.05
Mean	2.18	1.73	3.61	6.35
*P*-value	0.001		0.001	

ΔP=mean transvalvular gradient; cm^2^=square centimeters; EAO=effective orifice area; mm=millimeters; mmHg=millimeters of mercury

### Valve-in-valve in 31-mm bioprosthesis

For the bioprosthesis with a nominal size of 31 mm, tests were performed only with the 30-mm transcatheter valve. Thus, by associating the 31-mm mitral bioprosthesis with the 30-mm transcatheter valve, we found an EOA of 2.18 cm² (SD±0.04) and mean ΔP of 3.6 mmHg (SD±0.06), as shown in Table 6.

**Table 6:** Values found with the 31-mm bioprosthesis and the 30-mm transcatheter valve.

Hydrodynamic tests: Bioprothesis 31 mm × Inovare 30 mm		
	**EOA (cm^2^)**	**ΔP (mmHg)**
**Frequency**	**Inovare 30 mm**	**Inovare 30 mm**
70	2.1	3.7
80	2.2	3.6
90	2.2	3.6
100	2.2	3.5
110	2.2	3.6
120	2.2	3.6
Standard deviation	0.04	0.06
Mean	2.18	3.6

ΔP=mean transvalvular gradient; cm^2^=square centimeters; EAO=effective orifice area; mm=millimeters; mmHg=millimeters of mercury

## DISCUSSION

An increase in life expectancy and greater control of chronic diseases has contributed to the progression of the number of elderly patients with degenerated mitral bioprostheses, who usually present with multiple comorbidities. The surgical risk for new conventional valve replacement in patients under such conditions increases considerably.

Surgical valve replacement is the procedure of choice for patients with degenerated mitral bioprosthesis because of the satisfactory clinical results obtained over the years ^[9]^. The indication of mitral valve-in-valve becomes a new option for these patients at high surgical risk, because it tends to minimize the risk of mortality during the procedure ^[10]^.

The valve-in-valve implant technology for the treatment of prosthetic dysfunctions has been very attractive and has shown increasingly promising results in selected cases. It presents a series of consistent advantages ^[11]^, such as the radiopaque metallic ring of the degenerated bioprosthesis, present in most of these cases, which works as a perfect marker of the prosthesis release site, as a very well-visualized point and determined through the use of fluoroscopy.

In addition, the circular, rigid and symmetrical ring allows a more symmetrical anchorage, keeping the prosthesis firmly in place, with less risk of migration, compared to the transcatheter valve implantation in the native calcified mitral valve or in the native aortic ring (original indication of the transcatheter technique).

Among the benefits provided by this technique, we emphasize that the characteristics of the prosthetic ring also reduce the probability of occurrence of perivalve leakage in relation to the implant in the native valve, mainly because the ring is circular, unlike the native valve that has an implant area with a more asymmetrical shape and disproportionate calcifications in each leaflet ^[12]^.

Despite favorable published results, there is great concern regarding the reduction of EOA, due to the reduction of the transvalvular mitral gradient when compared to the implantation on native valves ^[13]^. In this context, it is imperative to perform tests with the scope of directing the limits of this therapy and to determine the best transcatheter valve size for each bioprosthesis, so that the patient will achieve the expected therapeutic benefit.

It is imperative for the surgeon to use the valve-in-valve technique for absolute knowledge of the bioprosthesis marking and size, because, although they have equal nominal diameters, different bioprostheses may have different internal diameters and true IDs, depending on how the bioprosthesis was made (i.e., whether the pericardial leaflets were made internally or externally to the bioprosthesis ring and its support posts).

Adequate expansion and anchoring of the transcatheter valve over another implanted in a bioprosthesis ring results from the correct oversizing between the true ID of the valve already implanted and the external diameter of the new valve. When oversizing is insufficient, valve migration may occur. On the other hand, if the oversizing is excessive, the valve will not show the desired expansion, especially of the pericardial leaflets, resulting in the appearance of higher transvalvular gradients in relation to the adequately expanded valve.

In valve-in-valve studies, the best results were obtained with oversizing between 10% and 20% ^[14]^. In our research, oversizing of the best combination results between bioprosthesis and transcatheter valve were also within the aforementioned parameter, as previously seen.

Since December 2010, there has been an international valve-in-valve procedures registry (VIVID International Data Registry), which provides analysis of the results obtained as well as guidelines for valve-in-valve procedures.

Three-dimensional echocardiography and tomography provide additional information necessary for correct measurement of the bioprosthesis. Such tools are particularly useful for situations in which there are no references regarding the previously implanted bioprosthesis and for those cases that do not have radiopaque markers for fluoroscopy.

Despite the encouraging results that have been published, it is important to clarify that there is still a great concern in relation to reducing EOA and increasing the transvalvar mitral gradient ^[15]^, reinforcing the need to perform such tests, with the purpose of guiding the limits of this therapy and reach the best transcatheter valve dimension for a given bioprosthesis.

The combination of results depends on the type of transcatheter prosthesis used and its individual constructive characteristics (i.e., size and height), as well as the amount of metal and pericardium used, and the bioprosthesis used (true ID and format).

In this scenario, the reported tests are included. Regarding the choice of the best transcatheter valve for a given mitral bioprosthesis, it was observed that the Inovare with better hydrodynamic performance for a given Braile bioprosthesis is 1 mm smaller than the nominal diameter. However, this fact is evident in the comparison with the oversizing of Inovare's external diameter and the true ID of the bioprosthesis when compared with the adequate oversizing reported in the literature.

Regarding the transcatheter valve-in-valve implant in a 27-mm mitral bioprosthesis, it should be weighted only in specific cases, preferably by a 26-mm transcatheter valve, as it has a better transvalvular gradient. The EOA is slightly larger, but still limited, when compared to the 28-mm transcatheter valve implant. Thus, the valve-in-valve in a 27-mm degenerated mitral bioprosthesis should be judicious and individualized for the patient in question.

Regarding the use of valve-in-valve in 29-mm and 31-mm degenerated bioprostheses, the transvalvular gradients are low with an adequate EOA. It should be noted that, for these bioprosthesis sizes, transcatheter valves of 1 mm smaller in dimension should be used, considering the demonstration of a better hemodynamic profile.

This is probably because the transcatheter valve is made in a chromium-cobalt wire structure and opened in the bioprosthesis ring by ballooning. With a larger transcatheter valve, this metallic structure would not open completely, hindering the proper performance of the prosthesis.

The mean transvalvular gradient and EOA data of the Valve-in-Valve International Data were: mean ΔP of 5.9±2.7 mmHg and EOA of 1.99±0.7 cm^2^. The data obtained by our transcatheter group (Gaia et al.) ^[11]^ were: mean ΔP of 11.1±5.0 mmHg and EOA of 1.73±0.54 cm^2^. Compared with our data, we found that these are in accordance with the values obtained in the real world.

The *in vitro* study, however, endures some limitations, such as the fact that the bioprostheses used are not degenerated, as we know that degenerated prostheses may present pannus (thickening) in their ring, which can reduce the true ID and not allow extrapolation. However, prospective evaluations of transcatheter implants *in vivo* allow gradient data and the expected EOA for a particular case, adjusting the case, if appropriate.

In addition, the implantation depth of the transcatheter prosthesis in a conventional biological prosthesis can determine different residual gradients, as demonstrated by Simonato et al. ^[15]^, for aortic position. In the mentioned study, it was demonstrated that there is an ideal point for implantation. In this study, only the position determined by the manufacturer's use indications was used. Future studies with different mitral implantation positions may show optimized results, especially in implants with the 27-mm bioprosthesis.

It is not possible to directly exorbitate these data for clinical practice, but this study suggests that the valve with the largest possible orifice area should be implanted at the time of the first valve replacement surgery. Thus, in case of bioprosthesis dysfunction, a valve-in-valve implant will be feasible, with less flow limitation and less pressure gradient generation, allowing, in exceptional situations, the implantation of multiple transcatheter implants (valve-in-valve-in-valve). As valve-in-valve implantations become routine and younger high-risk surgical patients require further surgery, the need for multiple valve-in-valve may become a reality.

The possibility of new hydrodynamic tests, but involving all other prostheses implanted in Brazil, would be ideal to allow the creation of a national guide for transcatheter valve implantation. Finally, through this study we can propose the creation of a national mitral valve-in-valve application, serving as a reference guide in choosing the most suitable size for this new technique in our country (Table 7).

**Table 7:** Recommendations on the best prosthesis to be used in the mitral valve-in-valve implant between the bioprosthesis and transcatheter valves of Braile Biomédica.

Valve-in-valve mitral (Inovare X Braile bioprosthesis)		
**Bioprosthesis**	**Inovare**	**Recommendation**
27 mm	26 mm	Use authorized for implant in individualized cases
29 mm	28 mm	Proper prosthesis, use authorized for implant
31 mm	30 mm	Proper prosthesis, use authorized for implant

mm=millimeters

## CONCLUSION

The valve-in-valve implantation of transcatheter valves in mitral bioprostheses proved to be feasible in *in vitro* testing. The use of 27-mm bioprostheses should be judicious, with preference for a 26-mm transcatheter valve, as it offers a better EOA and a smaller transvalvular gradient than the 28-mm transcatheter valve.

In the 29-mm and 31-mm bioprostheses, the implant was very satisfactory, with good EOAs and transvalvular gradients, with a preference for transcatheter valves 1 mm smaller, as a better hydrodynamic performance with statistical significance was observed. It was possible to prepare a recommendation guide for mitral VIV implantation for Inovare prosthesis in Braile biological prosthesis.

**Table:** 

Authors' roles & responsibilities	
TVN	Substantial contributions to the conception or design of the work; or the acquisition, analysis, or interpretation of data for the work; drafting the work or revising it critically for important intellectual content; final approval of the version to be published
CC	Substantial contributions to the conception or design of the work; or the acquisition, analysis, or interpretation of data for the work; drafting the work or revising it critically for important intellectual content; final approval of the version to be published
AB	Substantial contributions to the conception or design of the work; or the acquisition, analysis, or interpretation of data for the work; drafting the work or revising it critically for important intellectual content; final approval of the version to be published
HP	Substantial contributions to the conception or design of the work; or the acquisition, analysis, or interpretation of data for the work; drafting the work or revising it critically for important intellectual content; final approval of the version to be published
DG	Substantial contributions to the conception or design of the work; or the acquisition, analysis, or interpretation of data for the work; drafting the work or revising it critically for important intellectual content; final approval of the version to be published

## References

[1] Gammie JS, Sheng S, Griffith BP, Peterson ED, Rankin JS, O'Brien SM (2009). Trends in mitral valve surgery in the United States results from the society of thoracic surgeons adult cardiac surgery database. Ann Thorac Surg.

[2] Brown JM, O'Brien SM, Wu C, Sikora JA, Griffith BP, Gammie JS (2009). Isolated aortic valve replacement in North America comprising 108,687 patients in 10 years changes in risks, valve types, and outcomes in the society of thoracic surgeons national database. J Thorac Cardiovasc Surg.

[3] Naji P, Griffin BP, Sabik JF, Kusunose K, Asfahan F, Popovic ZB (2015). Characteristics and outcomes of patients with severe bioprosthetic aortic valve stenosis undergoing redo surgical aortic valve replacement. Circulation.

[4] Vandrecic MO, Gontijo Filho B, Oliveira SA, Paula e Silva JA, Fantini FA, Barbosa JT (1992). [Tratamento anticalcificante de bioprótese: resultado clínico inicial]. Rev Bras Cir Cardiovasc.

[5] Brandão CM, Pomerantzeff PM, Souza LR, Tarasoutchi F, Grimberg M, Oliveira SA (2002). [Fatores de risco para mortalidade hospitalar nas reoperações valvares]. Rev Bras Cir Cardiovasc.

[6] Bonhoeffer P, Boudjemline Y, Saliba Z, Hausse AO, Aggoun Y, Bonnet D (2000). Transcatheter implantation of a bovine valve in pulmonary position a lamb study. Circulation.

[7] Cribier A, Eltchaninoff H, Bash A, Borenstein N, Tron C, Bauer F (2002). Percutaneous transcatheter implantation of an aortic valve prosthesis for calcific aortic stenosis first human case description. Circulation.

[8] Dvir D, Webb JG, Bleiziffer S, Pasic M, Waksman R, Kodali S (2014). Transcatheter aortic valve implantation in failed bioprosthetic surgical valves. JAMA.

[9] Cheung A, Webb JG, Barbanti M, Freeman M, Binder RK, Thompson C (2013). 5-year experience with transcatheter transapical mitral valve-in-valve implantation for bioprosthetic valve dysfunction. J Am Coll Cardiol.

[10] Gaia DF, Braz AM, Simonato M, Dvir D, Breda JR, Ribeiro GC (2017). Mitral implant of the Inovare transcatheter heart valve in failed surgical bioprostheses a novel alternative for valve-in-valve procedures. Interact Cardiovasc Thorac Surg.

[11] Gaia DF, Couto A, Breda JR, Ferreira CB, Macedo MT, Gimenes MV (2012). Transcatheter aortic valve-in-valve implantation a selection change?. Rev Bras Cir Cardiovasc.

[12] Ionescu MI, Pakrashi BC, Holden MP, Mary DA, Wooler GH (1972). Results of aortic valve replacement with frame-supported fascia lata and pericardial grafts. J Thorac Cardiovasc Surg.

[13] Webb JG, Wood DA, Ye J, Gurvitch R, Masson JB, Rodés-Cabau J (2010). Transcatheter valve-in-valve implantation for failed bioprosthetic heart valves. Circulation.

[14] Paradis JM, Del Trigo M, Puri R, Rodés-Cabau J (2015). Transcatheter valve-in-valve and valve-in-ring for treating aortic and mitral surgical prosthetic dysfunction. J Am Coll Cardiol.

[15] Simonato M, Azadani AN, Webb J, Leipsic J, Kornowski R, Vahanian A (2016). In vitro evaluation of implantation depth in valve-in-valve using different transcatheter heart valves. EuroIntervention.

